# Occurrence of AH1N1 viral infection and clinical features in symptomatic patients who received medical care during the 2009 influenza pandemic in Central Mexico

**DOI:** 10.1186/1471-2334-12-363

**Published:** 2012-12-20

**Authors:** Juan Pablo Castillo-Palencia, Lucie Laflamme, Joel Monárrez-Espino

**Affiliations:** 1 Master in Public Health Program, San Luis Potosi Autonomous University, San Luis Potosi, Mexico; 2 Department of Epidemiological Surveillance, San Luis Potosi Health Services, San Luis Potosi, Mexico; 3 Division of Global Health (IHCAR), Department of Public Health Sciences, Karolinska Institutet, Nobels väg 9, 17177, Stockholm, Sweden

## Abstract

**Background:**

In 2009 a new influenza serotype (AH1N1) was identified in Mexico that spread rapidly generating worldwide alarm. San Luis Potosi (SLP) was the third state with more cases reported in that year. The clinical identification of this flu posed a challenge to medical staff. This study aimed at estimating the AH1N1 infection, hospitalization and mortality rates, and at identifying related clinical features in persons who received medical care during the influenza pandemic.

**Methods:**

Retrospective study with persons with flu-like illness who received public or private medical care in SLP from 15.03.09 to 30.10.09. Physicians purposely recorded many clinical variables. Samples from pharyngeal exudate or bronchoalveolar lavage were taken to diagnose AH1N1 using real-time PCR. Clinical predictors were identified using multivariate logistic regression with infection as a dependent variable. Odds ratios (OR) with 95% confidence intervals (CI) were computed. Analyses were stratified by age group based on the distribution of positive cases.

**Results:**

From the 6922 persons with flu symptoms 6158 had available laboratory results from which 44.9% turned out to be positive for AH1N1. From those, 5.8% were hospitalized and 0.7% died. Most positive cases were aged 5–14 years and, in this subgroup, older age was positively associated with A H1N1 infection (95% CI 1.05-1.1); conversely, in patients aged 15 years or more, older age was negatively associated with the infection (95% CI 0.97-0.98). Fever was related in those aged 15 years or more (95% CI 1.4-3.5), and headache (95% CI 1.2-2.2) only in the 0–14 years group. Clear rhinorrhea and cough were positively related in both groups (p < 0.05). Arthralgia, dyspnea and vaccination history were related to lesser risk in persons aged 15 years or more, just as dyspnea, purulent rhinorrhea and leukocytosis were in the 0–14 years group.

**Conclusion:**

This study identified various signs and symptoms for the clinical diagnosis of AH1N1 influenza and revealed that some of them can be age-specific.

## Background

Influenza A virus is a main cause of winter epidemics that results in increments in respiratory morbidity. It constitutes a public health concern due to the burden that it represents for the health system and labor market, and for its potential to evolve into a pandemic [1-3]. The virus is sub-classified based on its surface glycoproteins, hemaglutinin and neuroaminidase, which confer the pathogenicity [1,3-6]. Its high antigenic mutation capability is responsible for the cyclic outbreaks observed annually [1,5].

Transmission is mainly person to person through aerosol particles expelled by ill individuals when coughing or sneezing, but also through contact with hands or contaminated fomites [5]. The incubation period ranges from hours to days [5], with an average of two days [6]. Although some patients might be asymptomatic, most present signs and symptoms of varying severity after a few days of contracting the disease [6,7], but the majority usually recover within one or two weeks [1].

The first flu pandemic was recorded in 1538 [5]. In the last century three pandemics reached the equivalent of Category 2 on the CDC pandemic severity index (case-fatality ratio >0.1%), and all were attributed to the subtype A [1]: The Spanish flu (AH1N1) from 1918–1920 disseminated in Europe and the US causing about 50 million deaths [3,5,6,8]; the Asian flu (AH2N2) from 1957–1958, responsible for 1–4 million deaths, was caused by a viral mutation that resulted in a combination of avian and human strains; and the Hong Kong flu (AH3N2) from 1968–1969 that resulted from an antigenic shift caused nearly 1 million deaths [5,8].

The last pandemic started in Mexico in March 2009, when the Ministry of Health recognized an unexpected outbreak of respiratory disease [9] that would later be identified as AH1N1, a new viral strain resulting from a genetic combination of bird, pig and human influenza viruses [10]. The mutation detected in the nuclear M protein, responsible for the resistance to amantadine compounds, similar to that found in the AH5N1 Hong Kong virus from 1997, caused concern due to the high lethality seen with this kind of strain, and because of the high infectivity of this new virus [8].

By May, many countries started reporting cases, and as a result WHO defined the event of international importance [9], and governments implemented an emergency response plan to limit the viral spread and consequences [11]. In Mexico, educational and other non-essential activities were suspended for weeks to prevent people from getting infected [12]. By June 11, the WHO officially declared a pandemic [13].

Fortunately, this pandemic was less lethal than expected, resulting in about 18 500 deaths worldwide in 2009 [13]. In Mexico around 73 000 cases with nearly 1 350 deaths were confirmed, resulting in a lethality rate of ~1.8% [14,15]. San Luis Potosi (SLP) state, where this study was conducted, accounted for 6.1% of the cases registered in the country, in spite of having only 2% of the Mexican population (2.5 million) [14]. It was in fact the state with the third most cases reported [15].

The limited knowledge about the symptomatology associated with the presence of AH1N1 made it very difficult for physicians to clinically distinguish this flu virus from other respiratory infections. A better understanding of the association between symptomatology and the presence of the virus could assist in future diagnostic efforts.

This study estimated the AH1N1 infection, hospitalization and mortality rates in SLP during the 2009 pandemic, and aimed at identifying clinical features associated with AH1N1 infection in individuals with flu-like illness who sought medical care.

## Methods

### Study design

This study retrospectively investigated data gathered on patients suspected to be infected by AH1N1 virus during the 2009 outbreak and for which a set of clinical data was systematically collected.

Individuals of all ages with flu symptomatology who sought medical attention from 15.03.09 to 30.10.09 at any of the nearly 500 public or private health care facilities that integrated the Epidemiological Surveillance System for Influenza (ESSI) in the central Mexican state of SLP [16,17] were eligible for these analyses.

A working clinical definition was set during the pandemic to screen for all persons presenting fever, cough and headache [18,19], who were considered potentially infected, and respiratory tract samples were obtained to confirm the diagnosis; for infants irritability was used instead of headache, and in the elderly population fever could be missing [13,19]. In addition to these symptoms, many other clinical variables were systematically measured and recorded. In some cases, physicians decided to include patients for laboratory screening in spite of not having the pre-required three clinical manifestations.

### Data source

Data was extracted from the ESSI, administered by SLP State Health Services [16]. When a patient fulfilled the criteria for a probable case, physicians had to complete a specific questionnaire that included information about the patient’s basic socio-demographic characteristics, the unit where the health care was provided, the complete clinical symptomatology, the use of previous treatment, and data on specific epidemiological risk factors. These formats were then sent to one of the six health jurisdictions of SLP where data were coded and manually entered into the ESSI.

Samples from pharyngeal or nasopharyngeal exudate, or bronchoalveolar lavage were taken from all patients who fulfilled the operational definition to confirm the diagnosis of AH1N1 using real-time polymerase chain reaction (RT-PCR), which has been reported to have a very high sensitivity and specificity [20].

Specimens were placed in normal saline solution or viral transport media, and were kept cooled at 2-4°C [21] before analyses were carried out at the Public Health Laboratory of SLP and confirmed by the National Institute for Diagnostic and Epidemiological Reference in Mexico City, based on the primers designed by the CDC to detect the AH1N1 virus [22].

This study was carried out in compliance with the Helsinki Declaration. Potential ethical concerns derived from the use of human data were carefully considered; informed consent was not obtained as data was collected routinely for epidemiologic surveillance purposes. The proposal was reviewed and approved by the Ethics and Research Committee of SLP State Health Services (registration number SLP/070-2012) and authorization to use the data was provided by SLP State Health Services (approval reference number 19089).

### Data selection

For the purpose of this study, the clinical data extracted from the ESSI registry were recoded when necessary (e.g. tachypnea from respiratory rate) and classified into signs (i.e. objective medical characteristic that can be detected), symptoms (i.e. subjective feature noticed by the patient that cannot be directly observed), and epidemiological risk factors related to flu infection. Except for few continuous variables (e.g. age, fever or respiratory rate), most of these variables were coded dichotomously in the original questionnaire.

Variables were selected for the analyses if they were related to respiratory tract infections. However, some had to be excluded as they contained similar information (e.g. pharyngitis and sore throat) or because they were considered irrelevant for this study (e.g. contact with animals and recent travel). Complete data was available for all the clinical variables included.

The signs included were fever (arm temperature ≥38°C), cough, clear or purulent rhinorrhea, nasal congestion, sore throat, conjunctivitis, dysphonia, tachypnea (≥20 breaths/min; ≥40 for infants aged ≤5 years), cyanosis and leukocytosis (≥12000/μL); the symptoms included headache, malaise, chills, myalgia, irritability, arthralgia, thoracic pain and dyspnea; and the epidemiological risk factors included recent contact with persons with flu and history of antiviral treatment within two weeks prior to the interview, immunization against seasonal influenza, current smoking, and history of diagnosed asthma and chronic obstructive pulmonary disease.

### Statistical analyses

Infection, hospitalization and mortality rates were estimated per 100 individuals and defined as: Infection rate = Infected cases confirmed by laboratory / Total number of persons tested with available laboratory result; Hospitalization rate = Hospitalized with confirmed laboratory result / Total number of infected persons; and Mortality rate = Deaths with confirmed laboratory result / Total number of infected persons.

The proportion of persons with positive and negative AH1N1 infection was plotted by five-year age groups and compared with the distribution of the state population to identify the age categories with the highest incidence.

Individuals with positive and negative infection confirmed by laboratory were compared using crude odds ratios (OR) with 95% confidence intervals (CI) for every clinical feature under study.

Clinical predictors were identified using backward stepwise logistic regression with infection confirmed by laboratory as a dependent variable, and all clinical data as independent variables. Adjusted regression models were produced for the two age group categories defined: 0–14 years (children), and ≥15 years. All signs, symptoms, and epidemiological risk factors that showed significant crude ORs in the bivariate analyses were entered into the initial models. Cases with missing laboratory data were excluded from the multivariate analyses (n = 794). Only variables with significant adjusted ORs, as judged by the 95% CI, remained in the final models.

The accuracy of the model was evaluated by subtracting the proportional by chance accuracy rate (PCHAR) to the overall percentage of accurate predictions seen in the classification matrix of the final model. The accuracy was considered acceptable if a ≥25% increase was observed over the PCHAR. The model fit was assessed using the Hosmer-Lemeshow goodness-of-fit statistic. A good model fit was indicated by a non-significant Chi-square value (p > 0.05). All data was analyzed with IBM® SPSS® version 18 (IBM, New York, USA).

## Results

Table 1 presents the infection, hospitalization and mortality rates for AH1N1 in SLP compared with geographic regions of North America with documented statistics of the like [23-25]. Laboratory results were available for 89.9% of the 6922 persons screened who fulfilled the definition for probable case. Positive results lead to an infection rate of 44.9%. The hospitalization rate was markedly higher than the national rate (5.8 *vs.* 1.5%), but considerably lower than the American and Canadian figures (~14-15%). The mortality rate (0.7%) was very similar to the national and Canadian estimates (0.6 and 0.8, respectively), but notably lower than that reported in other geographic regions. Except for the mortality rates between Canada, Mexico and SLP, all other rates presented were statistically different (p < 0.01).

**Table 1:** AH1N1 influenza infection, hospitalization and mortality rates for individuals with flu symptoms who sought medical care by geographic area from March to October, 2009

**Geographic area**	**Screened with flu symptoms**^**1**^	**Laboratory result available**^**2**^	**Rate x 100 (n)**		
			**Infection**^**3**^	**Hospitalization**^**4**^	**Mortality**^**5**^
In the World^+^	-	-	(482 300)	-	1.2 (6 071)
American continent^+^	-	-	(185 067)	-	2.3 (4 399)
North America					
Canada^†^	-	-	(10 156)	15.7 (1 604)	0.8 (83)
USA^†^	-	134 899	42.7 (57 602)	14.2 (8 204)	1.9 (1 123)
Mexico^‡^	-	-	(50 234)	1.5 (800)	0.6 (328)
San Luis Potosi	6 922	6 158	44.9 (2 767)	5.8 (161)	0.7 (21)

^1^ Fever, headache and cough were compulsory (in infants irritability replaced headache; in elderly fever could be missing), but other symptoms could be present.

^2^ Nasopharyngeal exudate or bronchoalveolar lavage samples were taken at the moment of the physical exam; RT-PCR was used for diagnosis.

^3^ Infected cases confirmed by laboratory / total number of persons tested with available laboratory result.

^4^ Hospitalized with confirmed laboratory result / total number of persons infected.

^5^ Deaths with confirmed laboratory result / total number of persons infected.

^+^ Information up to November 1, 2009 [25].

^†^ Information up to October 31, 2009 [23].

^‡^ Information up to October 26, 2009 [24].

**Figure 1:**
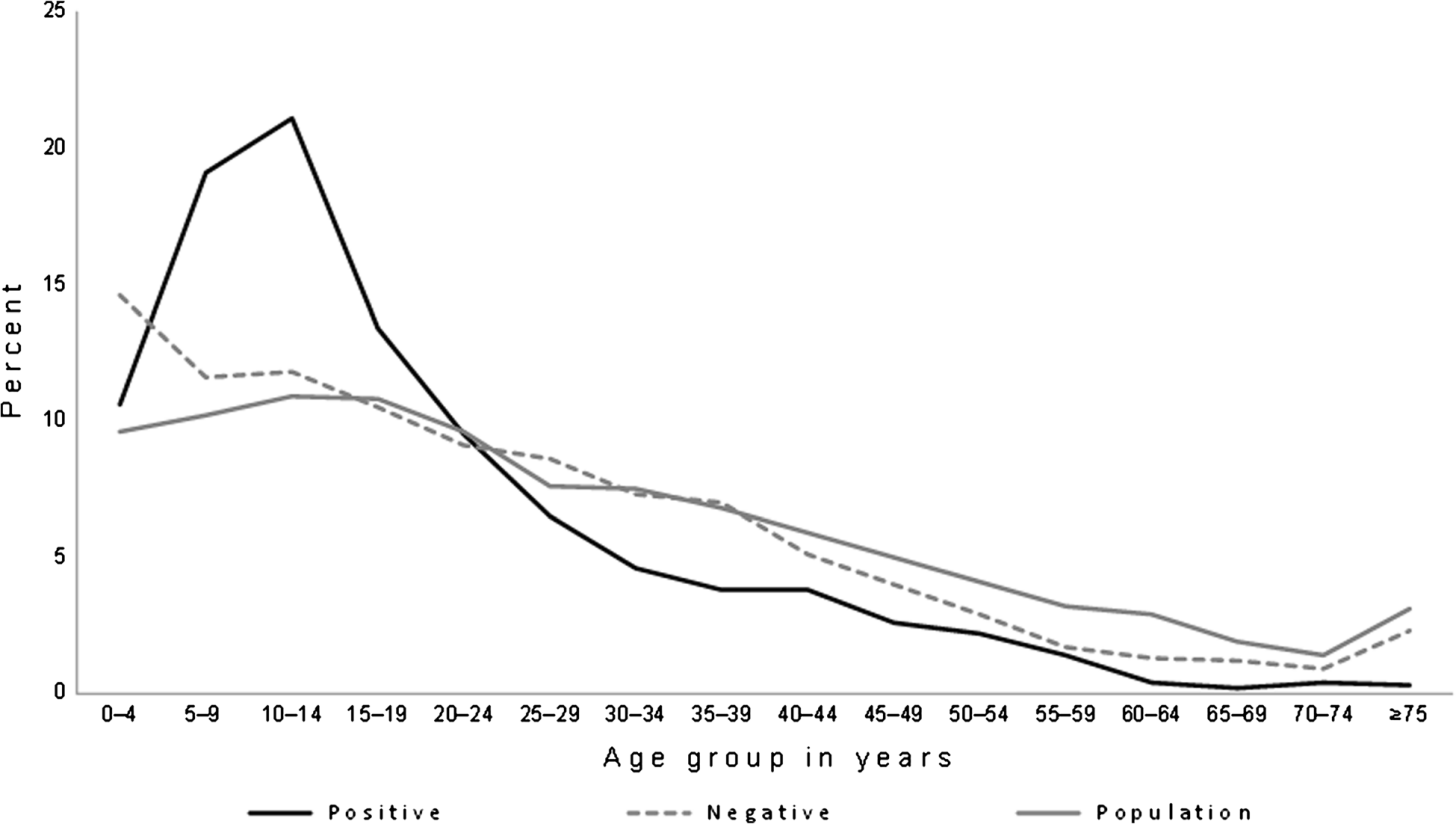
Distribution of individuals by age group with confirmed AH1N1 diagnosis who received medical care for flu symptoms from March to October 2009 compared with the population distribution of San Luis Potosi State, Mexico.

Figure 1 plots the percent distribution of confirmed cases by age group. From the 2767 patients with AH1N1 infection, 1409 (50.9%) were aged 0–14 years (731 males, 51.9%; 678 females, 48.1%), and 1358 (49.1%) were ≥15 years old (598 males, 44%; 760 females, 56%). There was a clear overrepresentation of positive cases (dark line) among children compared with the state population distribution (grey line). Consequently, the proportion of cases was lower in the adult groups compared with the population distribution, including the elderly.

Table 2 presents bivariate analyses for clinical manifestations associated with AH1N1 infection. Based on the crude associations (crude OR, 95% CI), most clinical signs and symptoms studied were significantly associated: The strongest positive associations with the infection were observed for fever (4.1, 3.3-5.1), cough (3.3, 2.7-4.0), headache (3.2, 2.6-3.8), and clear rhinorrhea (1.8, 1.6-2.1); and the strongest negative ones, with leukocytosis (0.13, 0.04-0.42), purulent rhinorrhea (0.56, 0.41-0.77) and dyspnea (0.57, 0.48-0.68). Sex was not related to infection (1.0, 0.9-1.1). Except for contact with other persons with flu (1.4, 1.2-1.7), all other epidemiological risk factors considered were not statistically associated.

**Table 2:** Clinical manifestations associated with AH1N1 influenza infection in individuals who received medical care in San Luis Potosi State from March to October 2009

**Clinical characteristics**		**Confirmed AH1N1 infection, n (%)**		**Crude OR [95% CI]**
		**Positive (n = 2767)**	**Negative (n = 3391)**	
Signs	Fever	2665 (96.3)	2927 (86.3)	4.14 [3.32–5.16]
	Cough	2610 (94.3)	2825 (83.3)	3.33 [2.76–4.00]
	Clear rhinorrhea	2071 (74.8)	2077 (61.3)	1.88 [1.68–2.10]
	Nasal congestion	1376 (49.7)	1361 (40.1)	1.47 [1.33–1.63]
	Sore throat	1498 (54.1)	1547 (45.6)	1.40 [1.27–1.55]
	Conjunctivitis	728 (26.3)	702 (20.7)	1.36 [1.21–1.54]
	Dysphonia	476 (17.2)	505 (14.9)	1.18 [1.03–1.36]
	Tachypnea	107 (3.9)	209 (6.2)	0.61 [0.48–0.77]
	Cyanosis	28 (1.0)	57 (1.7)	0.59 [0.37–0.94]
	Purulent rhinorrhea	57 (2.1)	122 (3.6)	0.56 [0.41–0.77]
	Leukocytosis	3 (0.1)	28 (0.8)	0.13 [0.04–0.42]
Symptoms	Headache	2591 (93.6)	2785 (82.1)	3.20 [2.68–3.82]
	Malaise	2087 (75.4)	2252 (66.4)	1.55 [1.38–1.76]
	Chills	1356 (49.0)	1423 (42.0)	1.32 [1.20–1.47]
	Myalgia	1775 (64.1)	1977 (58.3)	1.28 [1.15–1.41]
	Irritability	262 (9.5)	267 (7.9)	1.22 [1.02–1.46]
	Arthralgia	1570 (56.7)	1785 (52.6)	1.18 [1.06–1.30]
	Thoracic pain	449 (16.2)	548 (16.2)	1.00 [0.87–1.15]
	Dyspnea	218 (7.9)	439 (12.9)	0.57 [0.48–0.68]
Risk factors	Contact with infected (flu)	433 (15.6)	374 (11.0)	1.49 [1.29–1.73]
	Antiviral treatment^1^	47 (1.7)	48 (1.4)	1.20 [0.80–1.80]
	Seasonal flu vaccine	258 (9.3)	343 (10.1)	0.91 [0.77–1.08]
	Asthma/COPD	36 (1.3)	45 (1.3)	0.98 [0.63–1.52]
	Smoking	44 (1.6)	62 (1.8)	0.86 [0.58–1.28]

^1^ Includes mainly acyclovir, amantadine, ribavirin and rimantadine given within 2 weeks prior to the interview.

Table 3 presents the final logistic regression models by age group. For children, the model included six variables with statistically significant positive adjusted ORs (age in years 1.07, cough 1.5, headache 1.6, clear rhinorrhea 1.3, contact with flu person 1.6, and conjunctivitis 1.2), and three with negative adjusted ORs (dyspnea 0.51, purulent rhinorrhea 0.47, and leukocytosis 0.07). The model fitted well (p 0.11), and the accuracy was 25% more than the PCHAR (0.62 *vs.* 0.50). For those aged 15 years or more, three variables showed statistically significant positive adjusted ORs (fever 2.2, cough 1.7, and clear rhinorrhea 1.5), and four had negative adjusted ORs (age in years 0.98, arthralgia 0.81, dyspnea 0.73, and seasonal flu vaccine 0.73). The accuracy of the model was 24% more than the PCHAR (0.63 *vs.* 0.52), and the model also fitted well (p 0.72).

**Table 3:** Adjusted logistic regression models with clinical features associated with AH1N1 infection in persons with flu symptoms who received medical care in San Luis Potosi from March 15 to October 30, 2009, stratified by age group

**Variables included in the final model**	**0-14 years (n = 2698)**			**≥15 years (n = 3430)**		
	**Coefficient**	**Adjusted OR [95% CI]**	**P-value**	**Coefficient**	**Adjusted OR [95% CI]**	**P-value**
Age in years	0.07	1.07 [1.05-1.10]	0.000	−0.02	0.98 [0.97-0.98]	0.000
Fever	–	–	–	0.80	2.23 [1.41-3.54]	0.001
Cough	0.46	1.59 [1.12-2.24]	0.009	0.57	1.78 [1.19-2.67]	0-005
Headache	0.50	1.65 [1.21-2.25]	0.001	–	–	–
Clear rhinorrhea	0.26	1.30 [1.08-1.57]	0.005	0.41	1.50 [1.28-1.77]	0.000
Arthralgia	–	–	–	−0.20	0.81 [0.69-0.96]	0.014
Seasonal flu vaccine	–	–	–	−0.31	0.73 [0.57-0.93]	0.011
Contact with infected	0.52	1.68 [1.33-2.13]	0.000	–	–	–
Conjunctivitis	0.25	1.29 [1.07-1.56]	0.007	–	–	–
Dyspnea	−0.65	0.51 [0.35-0.75]	0.001	−0.31	0.73 [0.59-0.90]	0.003
Purulent rhinorrhea	−0.75	0.47 [0.28-0.78]	0.004	–	–	–
Leukocytosis	−2.56	0.07 [0.00-0.62]	0.017	–	–	–
Intercept	−1.61			−1.11		

## Discussion

An objective of this study was to estimate the AH1N1 infection, hospitalization and mortality rates in this representative population sample. We observed that 62.7% of the AH1N1 positive cases occurred in persons aged 0–18 years, which was very similar to the 60% reported from April 15 to May 5, 2009 in the US [10], and to the 61.8% national Mexican figure registered from March 11 to May 27, 2009 in those aged 0–19 years [2] (64.2% in this study). Based on a longer observation period, our study reinforces the observation that the AH1H1 virus affected mostly infant and adolescent populations.

The high infection rate seen indicated a rapid transmission of this virus, since nearly one out of two persons presenting respiratory symptoms such as fever, headache and cough had a confirmed laboratory diagnosis, and this supports the value of the operational definition used to screen individuals.

The hospitalization rate is more problematic to interpret and compare, as no data regarding the criteria used for hospitalization was available. While different viral pathogenicity and/or clinical severity cannot be completely ruled out, it seems that hospitalization criteria varied considerably across geographic areas, as indicated by the relatively large variation observed (5.8% in SLP, 1.5% in Mexico, and 14-15% in the US and Canada) [23].

The infection, hospitalization, and mortality rates can be compared with those of seasonal influenza in SLP during the same period, which were 5.3% (0-14y 3.7%, ≥15y 4.8%), 9.6% (0-14y 4.9%, ≥15y 12.5%), and 1.8% (0-14y 0.9%, ≥15y 2.4%), respectively [26]. While the seasonal infection rate contrasts with the much higher rate seen for AH1N1 (44.9%), the mortality rate is nearly 2.5 times higher (1.7 *vs.* 0.7%), pointing to the high infectivity, but low lethality of this virus, as previously observed [2,6].

The mortality rate was also similar to that of the Mexican (0.6%) and Canadian (0.8%) national estimates [23,24] though clearly lower than the US figure (1.9%). This difference is also difficult to explain, but it is unlikely to be due to underreporting in SLP, as health authorities mobilized most resources to track down any potential case of infection during the pandemic.

This study also looked at the association between clinical features and the presence of AH1N1 infection in persons who received medical care during the influenza pandemic in central Mexico, and the role played by age for the magnitude and direction of the association.

The main findings were that fever was associated, but only in those aged ≥15 years, while headache only in the 0–14 year group. Clear rhinorrhea and cough were positively related in both groups. Arthralgia, dyspnea and vaccination history were related to lesser risk in those aged ≥15 years, as dyspnea, purulent rhinorrhea and leukocytosis were in children.

The frequency of signs and symptoms found among infected individuals can be compared with that from other published studies. Fever, cough and sore throat were present in 96, 94, and 54%, respectively, compared to 94, 92 and 66% in the American Investigation Team [10]. According to the CDC morbidity and mortality weekly report (May 6, 2009) the most frequent clinical features reported in Mexico were fever (98%), cough (94%), rhinorrhea (83%), and headache (80%) [27]; these proportions were relatively similar to those found in SLP (96, 94, 75, 93%, respectively). However, comparisons must be treated with caution, especially with clinical signs, as diagnostic criteria used by physicians could have varied across studies. The results of this study are also in line with previous ones showing that AH1N1 infection was not sex specific [28-30].

Although several attempts have been made to relate clinical characteristics with the presence of the AH1N1 influenza virus, this study has three major strengths. A first merit lies in the size of the study population, which comprises the largest population-based sample (more than 6000 individuals) reported thus far. A second strength relates to the inclusion of both hospitalized subjects and outpatients of all ages. The third strength is related to the data treatment itself where multivariate regression analyses were used stratified by age group (0–14 and ≥15 years) defined based on the cut-off seen for the observed distribution of individuals with confirmed AH1N1 PCR-diagnosis.

The protective odds for infection found with seasonal vaccination against influenza in the adult population add to the controversy on this topic, as previous studies have reported protection [31-35], no effect [36-40], and even increased risk of infection [41]. While the potential cross-reactive protection of seasonal influenza vaccines through humoral and cell-mediated immune responses [42,43] needs further investigation, an upcoming review to assess the protection offered by influenza vaccines against circulating influenza A or B viruses that are not antigenically well-matched to vaccine strains will help elucidating this issue [44].

An additional finding is that clinical manifestations commonly related to bacterial respiratory infections, such as purulent rhinorrhea or leukocytosis, were negatively associated with the likelihood of positivity. This finding is similar to that reported by other authors. In a sample of 362 patients presenting with flu syndrome to an emergency unit in Spain, nasopharyngeal swabs were taken for AH1N1 detection with PCR; results showed that positive cases had significantly lower mean leucocyte counts, and the association remained in the multivariate logistic regression analysis when the lymphocyte count was used [45]. However, it is worth noting that purulent rhinorrhea could also result from a superimposed bacterial respiratory infection [6].

Similarly, the symptomatology associated with lower respiratory tract infections and its severity (e.g. cyanosis, dyspnea and tachypnea) was also related to protective odds. This finding might have to do with the time period between the onset of the symptoms and the visit to the health unit, as an indicator of the progression of the infection. However, no differences were seen in the mean number of days elapsed from the reported start of the symptoms to the health visit (~3 days) between those who had and did not have these symptoms (p > 0.05).

Very few clinical features might be relatively specific to flu viral infections [27,46,47], such as the presence of clear rhinorrhea that relates to the hemmagglutinin inhibiting effect on the surface glycoproteins that protect the respiratory tract cilia [4]. Respiratory diseases caused by influenza and parainfluenza viruses, adenoviruses or syncytial respiratory virus usually produce similar unspecific clinical symptomatology, characterized by fever, chills, malaise, headache, myalgia or cough [48]. In fact, a recent review of multivariate models devised to clinically diagnose influenza reported that only the combination of fever, cough and acute onset has a modest accuracy [49].

Compared with confirmed cases of seasonal influenza in SLP during the same period, those with AH1N1 infection aged ≥15 years showed significantly (p < 0.05) higher proportions of cough, clear rhinorrhea, nasal congestion, sore throat, malaise, chills, and myalgia (ranging from 8.3 to 16.4% higher), but had lower proportion of dyspnea (5.3% less); however, among those aged 0–14 years, the proportions were significantly higher only for cough (8.3%) and clear rhinorrhea (8.7%) [26].

For the AH1N1 influenza virus infection, various studies have not been able to identify symptoms that could predict the presence of AH1N1 infection using various designs and analytical procedures [50-52]. However, others have identified signs and symptoms associated to positive status. A retrospective study with 117 adult cases and 236 matched controls presenting with respiratory symptoms to hospital emergency departments in Toronto, Canada, showed higher associations for AH1N1 when various combinations of signs, symptoms and laboratory indicators were used, though only age, cough and fever remained associated in the multivariate analyses [53]. Another study with military personnel who visited primary health care clinics for febrile respiratory illness (i.e. fever, cough and sore throat) compared the signs and symptoms between those with positive and negative AH1N1 influenza virus using real-time PCR. Of the 2858 subjects recruited 821 were influenza cases, of which 434 were 2009 pandemic influenza AH1N1. The comparison of clinical features using multivariate logistic regression showed that sore throat, photophobia, injected pharynx and nausea/vomiting were negatively associated, while running nose, chills, fever and eye symptoms were positively related [54].

## Conclusion

This study estimated the frequency and progression on the AH1N1 influenza infection in symptomatic individuals, and was able to identify various associated clinical features that revealed the age specificity of several of them, indicating the importance of this factor when establishing the presumptive clinical diagnosis.

## Competing interests

The authors declare that they have no competing interests.

## Authors’ contributions

JPCP, LL and JME participated in designing the study. JPCP and JME performed the data analysis and drafted the manuscript. All authors participated in the interpretation of the results, and critically reviewed and approved the final manuscript.

## Pre-publication history

The pre-publication history for this paper can be accessed here:

http://www.biomedcentral.com/1471-2334/12/363/prepub
